# Prostate Artery Embolization as Minimally Invasive Treatment for Benign Prostatic Hyperplasia: An Updated Systematic Review

**DOI:** 10.3390/jcm13092530

**Published:** 2024-04-25

**Authors:** Corrado Ini’, Tiziana Vasile, Pietro Valerio Foti, Concetta Timpanaro, Davide Giuseppe Castiglione, Federica Libra, Daniele Falsaperla, Francesco Tiralongo, Francesco Giurazza, Cristina Mosconi, Emanuele David, Stefano Palmucci, Salvatore Lavalle, Massimo Venturini, Antonio Basile

**Affiliations:** 1 Radiology I Unit, University Hospital Policlinico “G. Rodolico-San Marco”, Via Santa Sofia 78, 95123 Catania, Italy; davidegiuseppecastiglione@gmail.com (D.G.C.); federica.libra@hotmail.it (F.L.); danielefalsaperla@gmail.com (D.F.); tiralongofrancesco91@hotmail.it (F.T.); 2 Department of Medical Surgical Sciences and Advanced Technologies “G.F. Ingrassia”—Radiology I Unit, University Hospital Policlinico “G. Rodolico-San Marco”, Via Santa Sofia 78, 95123 Catania, Italy; tiziana_vasile@hotmail.it (T.V.); pietrofoti@hotmail.com (P.V.F.); concettatimpanaro@gmail.com (C.T.); david.emanuele@yahoo.it (E.D.); spalmucci@sirm.org (S.P.); basile.antonello73@gmail.com (A.B.); 3 NANOMED-Research Centre for Nanomedicine and Pharmaceutical Nanotechnology, University of Catania, 95125 Catania, Italy; 4 Centro di Ricerca Multidisciplinare “Chirurgia delle Sindromi Malformative Complesse della Transizione e dell’Età Adulta” (ChiSMaCoTA), Department of Medical Surgical Sciences and Advanced Technologies “G.F. Ingrassia”, University of Catania, 95123 Catania, Italy; 5 Vascular and Interventional Radiology Department, Cardarelli Hospital, Via A. Cardarelli 9, 80131 Naples, Italy; francescogiurazza@hotmail.it; 6 Department of Radiology, IRCCS Azienda Ospedaliero—Universitaria di Bologna, 40138 Bologna, Italy; cristina.mosconi@aosp.bo.it; 7 Dipartimento di Medicina e Chirurgia, Università degli Studi di Enna “Kore” Cittadella Universitaria, 94100 Enna, Italy; salvatore.lavalle@unikore.it; 8 Diagnostic and Interventional Radiology Department, Circolo Hospital, Insubria University, 21100 Varese, Italy; massimo.venturini@uninsubria.it

**Keywords:** benign prostatic hyperplasia (C12.100.500.565.500, C12.200.294.565.500), embolization, therapeutic (E02.520.360, E02.926.500), transurethral resection of prostate (E04.950.774.860.625.750)

## Abstract

**Background/Objectives**: Benign prostatic hyperplasia (BPH) has a significant impact on the quality of life of symptomatic patients. In patients manifesting lower urinary tract symptoms (LUTS), prostatic arterial embolization (PAE) has become a topic of interest in recent years. The purpose of this systematic review is to analyze and review techniques and clinical outcomes of patients who underwent endovascular treatment of BPH, with a special focus on the comparison of surgical and endovascular procedures. **Methods**: Through the major scientific databases, 1225 articles were selected from the initial research utilizing specific keywords and medical subject headings. Based on the inclusion and exclusion criteria established for selecting relevant studies for our purposes, the systematic analysis of the literature was conducted on a total of seven articles. **Results**: We collected data on 718 male patients (408 patients underwent PAE and 310 received TURP). The technical success rate varied from 86% to 100% for TAE and was 100% for TURP. During a 12-month follow-up period, both PAE and TURP were comparable on the reduction in IPSS and QoL questionnaire, while TURP showed significant improvements in Qmax and in the reduction in prostate volume. Length and cost of hospitalization were lower for PAE. Complication and adverse events rates were higher in the TURP group rather than in the PAE group (60.6% vs. 35.5%). **Conclusions**: Prostatic artery embolization represents an emerging minimally invasive procedure for BPH. According to previously released clinical studies, quality-of-life and urological symptom scores of the PAE group were comparable to those of the TURP group. Our research strengthens the evidence supporting the effectiveness and safety of PAE as a therapy for LUTS related to BPH.

## 1. Introduction

Benign prostatic hyperplasia (BPH) is one of the most common diseases in elderly men, with an overall incidence rate ranging from 8.5 to 41 cases/1000 person-year and a prevalence rate of up to 80% in patients over 75 years old [1,2,3]. BPH is a multifactorial disease in which the hormonal set-up and lifestyle play a pathogenetic role. In BPH, prostate enlargement and modifications are due to a combination of stromal and glandular cell hyperplasia, mainly in the para-urethral transition zone. Usually, BPH has slow progression and patients have no significant symptoms at the initial stage of the disease. As the prostate hyperplasia progresses, patients manifest lower urinary tract symptoms (LUTS) which have a significant impact on their quality of life. Most patients can be managed conservatively with watchful waiting and laboratory exams and no therapy is needed unless BPH causes symptoms or complications (urinary tract infections, impaired kidney function, hematuria, stones, or urinary retention) [4]. Treatment of BPH aims to reduce urinary symptoms and improve the quality of life of patients. First-line therapy includes lifestyle changes and medical therapy with alpha-blockers, 5α-reductase inhibitors, muscarinic receptor antagonists, phosphodiesterase type 5 inhibitors, or vasopressin analogues. [4,5,6,7]. Surgical therapies like prostatectomy and transurethral resection of the prostate (TURP) have been considered as the gold standard treatment for BPH for several years. However, in the last year, different mini-invasive procedures have been proposed in order to reduce surgical complications, mainly in elderly patients. Prostatic arterial embolization (PAE) is an emerging minimally invasive procedure for the treatment of lower urinary tract symptoms caused by BPH in patients with contraindication to surgical therapies or in whom medical therapies fail to control symptoms, and it has been shown to have similar efficacy to traditional surgical techniques, with a lower risk of major adverse events [8]. The main indications for PAE are patients with moderate to severe LUTS (based on International Prostate Symptom Score (IPSS) and quality-of-life score) [9,10,11] who have not responded to conservative management or for whom medical therapy has been contraindicated; patients with contraindications to surgical procedures; sexually active patients who worry about retrograde ejaculation (a common side effect of TURP), erectile dysfunction, or urine incontinence which instead may benefit from PAE [12]; patients with a prostate volume > 30–50 mL. Therefore, providing a valid and safe minimally invasive treatment option is of great importance in patients with BPH.

The purpose of this article is to analyze and review common clinical practices and outcomes of patients who have undergone endovascular treatment of BPH, with a special focus on the comparison of TURP and PAE procedures.

## 2. Materials and Methods

The “PICO” and “Preferred Reporting Items for Systematic Review and Meta-Analysis (PRISMA)” criteria were followed to perform an extensive literature review in the field of PAE and comparison of TURP and PAE as effective treatments for BPH. We used the following medical subject headings (MeSH) and keywords, and systematically chose relevant search terms and gathered evidence for evaluation: “prostatic artery embolization”, “prostate embolization vs. TURP”, “TAE vs. TURP”, “prostatic artery embolization (PAE) and surgery”. The research included PubMed, Embase, Google Scholar, Cochrane Library, and Medline databases to find publications relevant to our review. No interval in the search period was specified. The search was performed from February 2023 to January 2024. The inclusion criteria were as follows: articles written in English and in which the entire content was accessible; the study population inclusion criteria were male patients with a diagnosis of BPH treated with TURP and PAE; men over 40 years old with LUTS (IPSS ≥ 8, Qmax < 15 mL/sec at non-invasive uroflowmetry); a minimum prostate volume of 20 mL determined by ultrasound or cross-sectional imaging examination. The exclusion criteria were recurring articles from the same authors; articles written in a language other than English and those whose entire content could not be accessed; case reports and case series articles; articles that were not compatible with the aims of our research due to the use of general keywords; studies that evaluated additional procedures other than PAE and TURP. The study population exclusion criteria were patients with pathologies other than BPH; patients with prostate cancer; patients with disorders of the central nervous system. In doubtful cases, articles were included in this stage named “uncertain” and required the evaluation of the full text to make the final decision. The abstract screening was performed using a pre-developed evaluation form applied to each study returned by the bibliographic search. This form contains the inclusion and exclusion criteria for each PICO or element defined in the protocol study (study type, patient population). First author, year of publication, types of study designs, number of patients enrolled, type of treatment, embolic material, technical and clinical success, complications, and outcomes consideration in a 30-day period of surveillance for PAE and TURP were all parameters analyzed for each study. Whenever possible, subgroups of patients undergoing treatment with specific embolic materials were indicated. The statistics on technical success, clinical success, and complications were also compared when available. Technical success was considered by achieving blockage of blood flow in the prostate arteries on angiography after embolization. Clinical success was quantitatively evaluated among studies through the following tests and scores: the International Prostate Symptom Score (IPSS), a seven-point symptom-related questionnaire and one question about quality of life (QoL) [9,10,11]; the International Index of Erectile Function (IIEF-5), a questionnaire investigating erectile dysfunction and identifying treatment-related responses; urodynamic test, which is a technique that evaluates how well the bladder and urethra hold and release urine [7,8,13,14]; the PSA value assessment and prostate volume quantification with ultrasound examination or other cross-sectional imaging. Post-PAE adverse event reporting has been systematized using the PAE-specific modification to the Clavien–Dindo grading system (I–IV) provided by Moreira et al. [15] (Table 1). This system was chosen to harmonize the conclusions reached by the authors of the many investigations and to highlight issues and whether they have clinical relevance.

**Figure 1:**
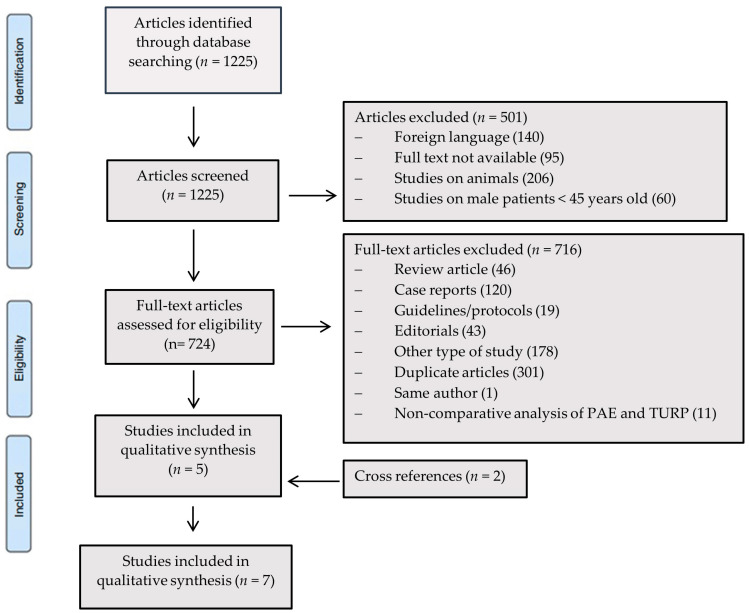
Flow-chart showing selection process of the articles included in the study.

Data were recorded using the Microsoft Excel database (Microsoft Corporate, version 2403, Redmond, WA, USA). First author, year of publication, types of study design, patients enrolled, and type of procedure were extracted from each study. Among the authors, two different radiologists (T.V. and C.I.) separately conducted the research for the studies, and differences over the literature data were settled through discussion among the other authors. For our research, we only considered studies that compared the clinical and technical success rates of TURP and PAE. A total of 1225 articles were selected from the initial research utilizing the previously mentioned keywords. Based on the aforementioned inclusion and exclusion criteria, the relevant articles for the purpose of our study were reduced to 5. A further 2 articles have been added analyzing cross-references from previous included studies. The systematic analysis of the literature was finally conducted on a total of 7 articles. The whole process of selecting studies based on the inclusion and exclusion criteria is summarized in Figure 1.

The study did not directly involve humans and did not require the Institutional Review Board approval of our department.

## 3. Results

**Figure 2:**
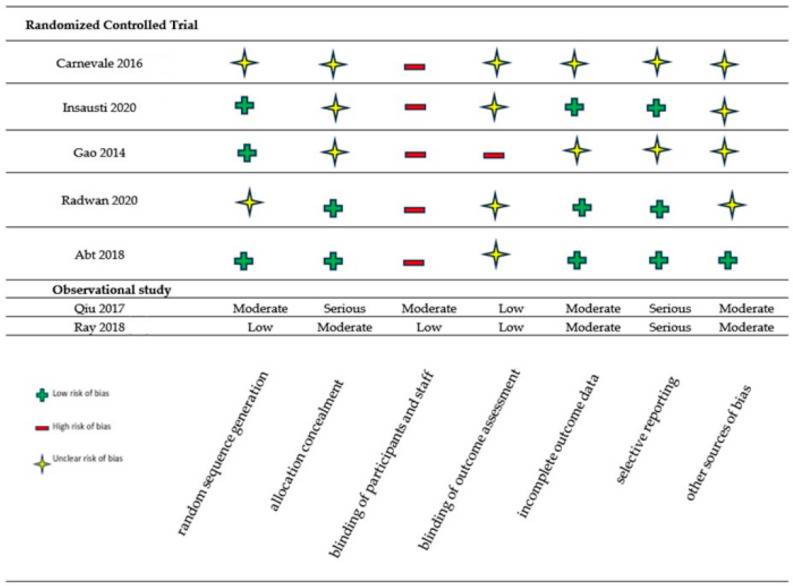
Risk of bias [16,17,18,19,20,21,22].

In the systematic review, we collected data on 718 male patients from seven different articles (five randomized controlled trials (RCTs), and two observational studies [16,17,18,19,20,21,22]) selected according to the inclusion and exclusion criteria. Using Cochrane’s risk of bias assessment tools, we evaluated the following bias risk domains: random sequence generation (selection bias), allocation concealment (selection bias), blinding of participants and staff (performance bias), blinding of outcome assessment (detection bias), incomplete outcome data (attrition bias), selective reporting (reporting bias), and other sources of bias. We judged risk of bias domains as “low risk”, “high risk”, or “unclear risk”. For selection bias (random sequence generation and allocation concealment), we assessed the risk of bias at the trial level. For performance bias (blinding of participants and staff), we considered all outcomes to be equally susceptible to performance bias. For detection bias (blinding of outcome assessment), we grouped outcomes as susceptible to detection bias (subjective outcomes) or not susceptible. We used the “Risk Of Bias In Non-Randomized Studies—of Interventions” tool, known as ROBINS-I, to assess the potential for bias in two research studies where randomization was not employed to assign interventions [21,22]. We judged risk of bias domains as “low risk”, “moderate risk”, and “serious risk”. An analytic summary of the risk of bias for all included studies is summarized in Figure 2.

A total of 408 patients underwent PAE and a total of 310 patients received TURP treatment. The mean age of patients was 67.4 years for PAE, and 68.1 years for TURP. All PAE procedures were conducted via a transfemoral arterial endovascular approach under local anesthesia. Five trials used the classic technique to perform the PAE, while in two studies, the Proximal Embolization First, Then Embolize Distal (PErFecTED) technique was performed. In all cases, microparticles and microspheres (size range 90–500 µm) were used as permanent embolizing material, except for one study in which the embolizing material was not specified. TURP was performed under spinal or general anesthesia; two studies used the monopolar TURP (M-TURP) technique, three studies used the bipolar TURP (B-TURP) technique, and in two studies, both bipolar and monopolar techniques were compared. Six out of seven studies reported procedural characteristics and other intra- and peri-procedural data (mean fluoroscopy time and mean procedure duration). The mean procedure duration was 116.2 min for PAE and 69.7 min for TURP, with a standard deviation of 29.3 and 9.8, respectively. The mean fluoroscopy time in PAE procedures was reported in four study with a mean time of 44.1 min. Hospital stay (days) was analyzed in four studies and the mean length of hospitalization was 1.7 days, with a standard deviation of 0.9 for PAE, and 3 days, with a standard deviation of 1.7 for TURP. This allows for a significative reduction in costs of hospitalization for patients treated with PAE. The main data extracted from the selected studies are summarized in Table 2.

Post-operative outcomes and complications were evaluated during the follow-up period. Two studies reported both short-term and long-term follow-up outcomes (up to 24 months), while five studies reported only short-term follow-up outcomes (up to 12 months). Technical success was analyzed in five out of seven studies. The technical success rate varied from 86% to 100% for TAE, with a mean value of 95.6%, and was 100% for TURP. In the seven studies included, all primary and secondary clinical outcomes were reported, and at pre-operative baseline, PAE and TURP groups were comparable with respect to age, prostate volume, Qmax, IPSS, and QoL. Three studies also evaluated the IIEF parameter, remaining unchanged among the two groups before and after procedures. After the 12-month follow-up period, both PAE and TURP were comparable on the reduction in IPSS and QoL questionnaire; TURP instead showed significant improvements in Qmax parameters. In six out of seven studies, prostate volume (mL) was evaluated before and after endovascular or surgical treatment. The mean prostate volume at baseline was 68.2 mL, with a standard deviation of 17.1 for the PAE group, and 61.5 mL with a standard deviation of 6.1 for the TURP group. The mean prostate volume after treatment was 47.8 mL, with a standard deviation of 13.2 for the PAE group, and 27.9 mL with a standard deviation of 6 for the TURP group. Therefore, the reduction in prostate volume was greater in patients who underwent TURP. Among the studies, different classification systems were used to categorize adverse events and complications [23]. A total of 145 adverse events and complications were identified in patients who underwent PAE and a total of 188 adverse events were identified in patients treated with TURP. Complication and adverse events rates were higher in the TURP group rather than in the PAE group (60.6% vs. 35.5%). The most common complication detected after PAE was local pain and acute urinary retention, while after TURP, it was hematuria. The number and type of complications reported in the individual studies are collected in Table 3.

## 4. Discussion

Benign prostatic hyperplasia is a benign enlargement of the prostate gland caused by an uncontrolled hyperplastic proliferation of the epithelial and fibromuscular tissues of the transition zone and periurethral area. As a consequence, the urethra is compressed by hyperplastic nodules, which results in a mechanical blockage of urine outflow and increased resistance that irritates the bladder detrusor muscle. This condition leads to the development of lower urinary tract symptoms divided into storage and voiding symptoms. BPH represents a common disease in elderly male patients, and it is also burdened by considerable social and economic costs which pay attention to treatment cost savings among procedures. The widespread mini-invasive procedure, mainly in elderly patients, allows the treatment of patients with contraindications to surgeryor with multiple comorbidities, achieving a high technical and clinical success rate. The history of prostate artery embolization is quite recent. In 1977, Bischoff first described a case of endovascular treatment applied to prostatectomy-related bleeding [24]. PAE was first tested for the treatment of BPH on animals in 2008 and 2009, respectively, on pigs and dogs [25,26], while it was only applied for the treatment of BPH-related symptoms in humans in 2010 [27]. The first study comparing PAE and TURP was published by Gao et al. in 2014 [18] and since then, few scientific studies have addressed this topic. In the present review, we included seven clinical studies comparing the short and long-term follow-up outcomes among patients who underwent TURP versus PAE for the treatment of BPH. Little research comparing PAE and TURP was accessible, especially as only studies that assessed both PAE and TURP in the same analysis were eligible for the inclusion criteria we established.

The aim of artery embolization is to devascularize the prostate. The embolization of the prostatic arteries determines an ischemic effect which reduces the size of the prostate gland, and therefore, the symptoms. Furthermore, smooth muscle relaxation may result from a decrease in the density of α-1 adrenergic receptors in the embolized prostate, which could enhance this effect [28,29]. Embolization of at least half of the prostate ranged from 90% to 98% in almost all articles published on this topic. Bilateral embolization should always be the end goal of PAE since it produces better clinical outcomes, greater primary treatment success rates, less symptom recurrence, and a lower rate of re-treatment. The CIRSE society defined the clinical success and failure criteria after PAE in December 2019 [30]. An International Prostate Symptom Score (IPSS) of less than 18, a score which decreases of at least 25%, a quality-of-life score of less than or equal to 3, and at least a one-point decrease from the baseline are considered the main criteria for symptomatic improvement after PAE. On the other hand, the persistence of severe symptoms or a reduction in peak urine flow are considered a clinical failure of the operation. Comparing the studies regarding intra- and peri-procedural data, the mean procedural time was longer for PAE rather than for TURP [17,18,19]. Mini-invasive procedures and endovascular treatments require an intraoperative analytic preliminary study of vessels supplying the prostate in extending the procedural time more than surgical treatment. In fact, pelvic arterial supply is significantly interconnected by anastomoses, the majority of which have modest flow characteristics and are only visible on angiograms with a pressurized contrast media injection [31]. Some anastomoses allow for contact between the prostate area and various clinically significant anatomical structures, such as the bladder, rectum, and penis. Since variations in anatomy are also frequently present, an analytic preliminary angiographic study is fundamental to prevent non-target embolization [32]. The assessment of clinical outcomes was mainly based on short-term evidence (up to a 12-month follow-up) from both randomized controlled trials and non-randomized studies. Regarding urologic symptom scores and quality-of-life questionnaires, no significant differences were detected between the two procedures in improving short-term urologic symptoms. In patients treated with TURP, Qmax and prostate volume were slightly improved rather than PAE, in all studies; a possible explanation of these data is that the embolization of prostatic vessels requires several months in reducing volume prostate, since ischemic effect and smooth muscle relaxation resulting from a decrease in the density of α-1 adrenergic receptors are a chronic process [28,29]. Erectile function exploring the IIEF-5 score showed only a slight decrease after both treatments, which did not differ significantly between treatments [20]. However, even if there was no change in erectile dysfunction between the two procedures, according to four studies analyzed, PAE could reduce problems with ejaculation [16,17,20,22]. In a long-term analysis conducted by Abt et al., retrograde ejaculation was present in 56% of patients who underwent PAE and in 84% of patients who underwent TURP, increasing patient treatment satisfaction [20]. Finally, our systematic review confirms that when TURP and PAE are compared in relation to short-term follow-up, patients appear to have equivalent improvements in their quality of life and urological symptoms after treatment.

The focus on reducing treatment-related complications is an important endpoint of modern studies comparing different strategies of treatment. All PAE procedures were conducted under local anesthesia, reducing costs and hospital stays of patients compared to TURP, since surgical procedures required spinal or general anesthesia and longer post-procedure observation. The shorter hospital stays for PAE represents a significant benefit of mini-invasive procedures. Furthermore, patients with serious comorbidities who cannot undergo general anesthesia might receive treatment for BPH thanks to the local anesthetic procedures. In 2017, Bagla et al. investigated the costs associated with TURP and PAE in a real-world United States of America hospital setting, discovering that direct costs of PAE were substantially lower rather than TURP; this was related to differences in the duration of stay, anesthetic, and staff members [33]. The use of ionizing radiation in PAE, unlike TURP, could represent a risk factor for exposure-related pathologies but no adverse effects related to fluoroscopy are mentioned in any study, maybe due to a short observational period. The analysis of the complications of the two procedures took into account both major and minor complications. However, there is no uniformity among the data collected, as the various studies used different classifications of adverse events, and some did not mention them, making the types of minor and major adverse occurrences ambiguous. Gao et al. showed that following PAE, complications were more common, while Ray et al. demonstrated that the PAE technique has a low complication rate, examining a larger population of patients [22]. Although the data collected are not homogeneous, complication and adverse events rates were higher in patients who underwent the TURP procedure, and post-procedural hospitalization was significantly reduced in patients who underwent PAE. Most PAE-related adverse effects are minor and are comparable to those from other endovascular embolization procedures [22,34]. In a review, Moreira et al. distinguished two groups: complications, which are any unexpected bad treatment-related outcomes, and side effects, which are any predicted yet unfavorable responses [15]. Adverse events can also be divided into intraoperative and postoperative events. Intraoperative side effects include drug or contrast material responses, vascular access accidents, device failure or incompatibility, unsuccessful catheterization and embolization techniques, manufacturing flaws, or incorrect material use. Acute urine retention, dysuria, hematuria, rectal bleeding, hematospermia, and urinary infection are considered temporary post-PAE side effects. Postembolization syndrome represents another expected side effect characterized by signs and symptoms such as nausea, vomiting, mild heat, painful urination, pelvic pain, rectal bleeding, and hematuria [35]. In the first two days following PAE, patients could also experience a small pressure or mild pain in the pelvic area that radiates into the perineal area. With oral analgesics, these problems can be effectively controlled. Major complications include non-target embolization, which can result in bladder or gland ischemia, and urinary sepsis which may necessitate readmission for intravenous antibiotic therapy.

In the literature, different studies tried to perform a meta-analysis on the comparisons of outcomes and clinical and technical success of the two procedures. Jiang et al. first elaborated on a meta-analysis to compare the efficiency and safety of TURP and PAE for the treatment of BPH in 2019 [36]; they analyzed four studies concluding that TURP was more effective than PAE in treating BPH, although PAE represents a safe and effective technique that improves the urodynamic parameters and quality of life of patients [16,18,21,22]. In their meta-analysis, Knight et al. support the therapeutic advantage of PAE, claiming that PAE and TURP showed comparable improvements in patient-reported symptoms, while TURP showed greater improvement in several objective BPH measures evaluated. Compared to TURP, PAE was also linked to less adverse events. In 2022, Jung et al. published a recent meta-analysis on both short- and long-term follow-up outcomes, concluding that the effects on urologic symptoms and patient-perceived improvements in quality of life seem to be comparable in the PAE and TURP group [37]. Our results confirm and are in agreement with previous studies reported in the literature [38,39,40].

The latest guidelines of the American Urological Association (AUA) for the management of BPH have included PAE in the armamentarium of the therapies of BPH, as reported by Bilhim et al. in a recent publication of November 2023 [41,42]. This important update, born from collaboration between urologists and interventional radiologists, states that PAE could be a valid alternative to other long-term medical therapies or more invasive urologic surgery, presenting different advantages, first of all because it requires minimal sedation, then avoids trans-urethral access and preserves sexual and ejaculatory functions. The inclusion of prostate artery embolization in the American urological guidelines validates and confirms the safety and efficacy of PAE in treating BPH, as reported in our systematic review.

## 5. Limitations

Our systematic review presents different limitations. The inability to include the duration of treatment effectiveness in the analysis due to a lack of outcome data significantly limits our study. Although several longitudinal trials have provided information about the length of improvement for TURP, long-term data for PAE are only now starting to surface, with preliminary results indicating a similar duration of efficacy for TURP [16,18]. However, there is still uncertainty regarding the rate of re-intervention in the PAE group [43]. As previously mentioned, the main limitation of this review was the relatively small number of studies comparing PAE and TURP that were accessible for our research. Analysis of the data collected was mainly based on both randomized controlled trials and non-randomized studies, and some data were extrapolated from a “mixed” series, representing this a limit in terms of the scientific evidence. Despite being conducted in Asia, Europe, and America Latina, all the included studies were carried out at single-center locations. Furthermore, the great variability in number, mean age, and comorbidities of patients makes data inhomogeneous. Regarding treatment techniques, the extrapolated data were variable. A different TURP approach (bipolar or monopolar) was utilized as a comparator in some included studies. Studies comparing bipolar TURP to monopolar TURP may exaggerate the risk of adverse events because bipolar TURP is known to have a lower rate of adverse events. Variability regarding the embolization procedure was also present: in some studies, the standard PAE technique was used, in others, the PERFECTED technique was used; in one study, the PAE and PERFECTED techniques were compared, and in some studies, the adopted embolization technique was not fully described. There was also great variability in the presentation of primary and secondary outcomes and adverse events among various studies comparing PAE and TURP.

## 6. Conclusions

Over the last 20 to 30 years, there has been a significant change in the treatment of BPH and related LUTS, with a greater emphasis on medical therapy and improved primary care management contributing to this transformation. TURP and, more recently, laser prostatectomy, like HoLEP, have historically been offered to patients who reach the point where they need an interventional procedure. Prostatic artery embolization has recently been represented as an alternative effective and safe treatment in male patients who cannot undergo TURP or for whom surgical therapies have been contraindicated, with a positive 5-year outcome: embolization of the gland can result in reduced volume without the need for surgical tissue removal. Despite the limitations, results of our systematic review agree with the latest meta-analyses on this topic [38,39,40]. According to previously released clinical studies, quality-of-life and urological symptom scores of the PAE group were comparable to those of the TURP group. With clinical results equivalent to TURP, this research strengthens the evidence supporting the effectiveness and safety of PAE as an additional therapy for LUTS related to BPH. Additionally, many patients with surgical comorbidities who were previously only able to receive supportive care now have a new option for treating LUTS, since PAE requires only mini-invasive access and local anesthesia. Further studies should continue by comparing larger numbers of patients with long-term follow-up to assess the longevity of the appropriate method and evaluate its cost effectiveness.

## Figures and Tables

**Table 1:** Clavien–Dindo classification of surgical complications adapted to PAE [15].

Grade	Definition
I	Any unexpected deviation from the normal post-embolization course without the need for additional pharmacological, urologic surgical/endoscopic, or radiological procedures.
II	The need for pharmacological treatment with drugs other than those allowed for grade I, as therapeutic use of antibiotics due to infection. Indwelling catheters are used in case of early acute urinary retention. Additional non-invasive tests.
III	The need for pharmacological treatment with drugs used in grade II, as well as surgical/endoscopic or radiological procedures, under or without general anesthesia.
IV	Any deviation from the normal post-embolization course with a life-threatening complication requiring ICU-management due to single or multi-organ dysfunction.
V	Death
Suffix ‘d’	The suffix “d” (for disability) is added to the complication grade if the patient has a complication at the time of discharge, indicating the need for a follow-up to fully evaluate the complication

**Table 2:** Main characteristics of the seven selected studies.

First Author, Year	Type of Study Design	Number of Patients	Embolic Material (PAE)	Mean Procedure Time (min)	Mean Fluoroscopy Time (min)	Age Mean (SD)	Follow-up (Months)	Mean Hospital Stay (Days)
Insausti, 2020 [17]	Randomized controlled trial	PAE (23) B-TURP (22)	300–500 µm of polyvinyl alcohol microspheres	PAE 138.7 TURP 70.2	PAE 58.0 TURP N/A	PAE 72.4 (6.2) TURP 71.8 (5.5)	12	PAE 1 TURP 1
Abt, 2021 [20]	Randomized controlled trial	PAE (48) M-TURP (51)	250–400 µm embozene microspheres (Boston Scientific, Boston, MA, USA)	Not collected	Not collected	PAE 65.7 (9.3) TURP 66.1 (9.8)	24	PAE 2.2 TURP 4.2
Carnevale, 2016 [16]	Randomized controlled trial	PAE (15) PERFECTED (15) M-TURP (15)	300–500 µm tris-acryl gelatin microspheres (embosphere microspheres; Merit Medical, South Jordan, UT, USA)	PAE 144.8 PERFECTED 147.5 TURP 61.7	PAE 49.2 PERFECTED 45.8 TURP N/A	PAE 63.5 (8.7) PERFECTED 60.4 (5.2) TURP 66.4 (5.6)	12	PAE not mentioned PERFECTED 6 hours TURP 2.1
Gao, 2014 [18]	Randomized controlled trial	PAE (54) B-TURP (53)	Polyvinyl alcohol microspheres (355–500 µm, Ivalon; Cook, IL, USA)	PAE 89.7 TURP 83.5	PAE 33.2 TURP N/A	PAE 67.7 (8.7) TURP 66.4 (7.8)	1–3–6–12–24	PAE 2.9 TURP 4.8
Radwan, 2020 [19]	Randomized controlled trial	PERFECTED(20) M-TURP(20) B-TURP (20)	Microspheres	PAE 89 M-TURP 59 B-TURP 68	PAE (Not collected) TURP N/A	PERFECTED 63 TURP 63	1–6	Not mentioned
Ray, 2018 [22]	Observational study	PAE (216) M-TURP(45) B-TURP (44)	-	PAE 144 TURP (Not collected)	PAE 38 TURP N/A	PAE 66 (7.4) TURP 70 (7.5)	1–3–6–12	Not mentioned
Qiu, 2017 [21]	Observational study	PAE (17) B-TURP (40)	Embosphere microspheres (90–180 µm; Merit Medical, Rockland, MA, USA)	PAE 90 TURP (Not collected)	PAE (Not collected) TURP N/A	PAE 75.35 (4.74) TURP 73.35 (4.75)	3–6–12	Not mentioned

**Table 3:** Complication rates and adverse effects after PAE and TURP.

Fist Author, Year	Number of Patients	PAE Complications (Mean Rate)	TURP Complications (Mean Rate)
Insausti, 2020 [17]	PAE (23) B-TURP (22)	Grade I * 26.7% Grade II * 73.3% Grade III * 0.0%	Grade I * 53.2% Grade II * 44.7% Grade III * 2.1%
Abt, 2021 [20]	PAE (48) M-TURP (51)	Grade I * 66% Grade II * 24% Grade III * 11%	Grade I * 64% Grade II * 22% Grade III * 15%
Carnevale, 2016 [16]	PAE (15) PERFECTED (15) M-TURP (15)	•Transient minimal rectal bleeding (6.7% in each group) ** •Hematospermia (6.7% in each group) ** •Reduction in ejaculate volume (13.3% in PAE group, 6.7% in PERFECTED group) ** •Transient pubic bone ischemia (6.7% in PAE group) ** •Hematuria (13.3% in PAE group) **	•Pollakuria, dysuria, and hematuria (100%) ** •Intra-operative damage to the left venous sinus and rupture of the prostatic capsule (6.7%) ** •Hematuria (6.7%) ** •Early urinary incontinence (26.7%) ** •Retrograde ejaculation (100%) ** •Prostate cancer identified incidentally (6.7%) **
Gao, 2014 [18]	PAE (54) B-TURP (53)	Minor advent * 40.7% Major advent * 14.8%	Minor advent * 24.5% Major advent * 7.54%
Radwan, 2020 [19]	PERFECTED (20) M-TURP (20) B-TURP (20)	•Acute urinary retention following catheter removal (10%) •Postembolization syndrome (20%) •Severe perianal pain (20%)	Not mentioned
Ray, 2018 [22]	PAE (216) M-TURP (45) B-TURP (44)	•Hematuria 18.6% •Hematospermia 12.6% •Incontinence 1.0% •Urinary infection 5.0% •Retrograde ejaculation 24.1%	•Hematuria 63.9% •Hematospermia 1.6% •Incontinence 3.3% •Urinary infection 1.6% •Retrograde ejaculation 47.5%
Qiu, 2017 [21]	PAE (17) B-TURP (40)	•Hypogastralgia (pain in the perineum, retropubic space, and/or urethra) 17.6% •Fever 29.4%	Not mentioned

* Grading according to Clavien–Dindo Classification System. ** Grading according to National Cancer Institute Common Toxicity Criteria for Adverse Events, version 4.0.

## Data Availability

The data presented in this study are available on request from the corresponding author.
